# Neuroimmune expression in hip osteoarthritis: a systematic review

**DOI:** 10.1186/s12891-017-1755-2

**Published:** 2017-09-11

**Authors:** Manuel Ribeiro da Silva, Daniela Linhares, Daniel Marques Vasconcelos, Cecilia Juliana Alves, Nuno Neves, Gilberto Costa, Meriem Lamghari

**Affiliations:** 1 i3S - Instituto de Investigação e Inovação em Saúde, Universidade do Porto, Rua Alfredo Allen, 4200-135 Porto, Portugal; 2 INEB - Instituto de Engenharia Biomédica, Universidade do Porto, Porto, Portugal; 3 Serviço de Ortopedia e Traumatologia, Centro Hospitalar São João, Porto, Portugal; 4 Faculdade de Medicina, Universidade do Porto, Porto, Portugal; 5 Orthopedic Department, CHSJ - Centro Hospitalar de São João, Alameda Prof. Hernâni Monteiro, 4200-319 Porto, Portugal; 6 MEDCIDS - Faculdade de Medicina, Universidade do Porto, Porto, Portugal; 7 ICBAS - Instituto Ciências Biomédicas Abel Salazar, Universidade do Porto, Porto, Portugal

**Keywords:** Hip osteoarthritis, Neuroimmunomodulation, Inflammation, Cytokines

## Abstract

**Background:**

Neuroimmune axis is central in the physiopathology of hip osteoarthritis (OA), but its specific pathways are still unclear. This systematic review aims to assess the nervous and immune system profile of patients with hip osteoarthritis (OA) when compared to healthy controls.

**Methods:**

A systematic review followed PRISMA guidelines was conducted. A two-step selection process was completed, and from 609 references 17 were included. The inclusion criteria were: original articles on adult patients with hip OA, with assessment of neuroimmune expression. Articles with other interventions prior to analysis and those without a control group were excluded.

**Results:**

Thirty-nine relevant neuroimmune markers were identified, with assessments in bone, cartilage, synovial membrane, synovial fluid, whole blood, serum and/or immune cells. GM-CSF, IFN-γ, IL-1α, IL-6, IL-8, IL-1 and TNF-α presented variable expression among tissues studied when compared between hip OA and controls. VEGFs and TGF-ß isoforms showed similar tendencies among tissues and studies. On nervous expression, CGRP, Tuj-1 and SP were increased in synovial membrane. Overall, patients with hip OA presented a higher number of overexpressed markers.

**Conclusions:**

For the first time a systematic review on neuroimmune expression in patients with hip OA found an upregulation of neuroimmune markers, with deregulated balance between pro and anti-inflammatory cytokines. However, no clear systematic pattern was found, and few information is available on nervous expression. This highlights the importance of future research with clear methodologies to guide the management of these patients.

**Electronic supplementary material:**

The online version of this article (10.1186/s12891-017-1755-2) contains supplementary material, which is available to authorized users.

## Background

Hip osteoarthritis (OA) is a common chronic health condition and a leading cause of pain and disability among adults, impacting many health outcomes [1]. The complex and multifactorial nature of hip OA is nowadays under the spotlight, and recent studies proposed a switch of the paradigm from a simple “wear and tear” to a much more complex mechanism, in which inflammatory mediators play a pivot role in initiation and progression of the pathologic process [1, 2].

Neuroimmune axis is known to control the development and perpetuation of multiple inflammatory diseases [1, 3]. Immune cells and secreted cytokines have been established as important players in OA [4]. Also, neuropeptides were recently proposed as critical molecules in the modulation of the inflammation and pain associated with OA [5]. Recent works showed that each joint should be seen as an individual organ, with OA being not exclusively a disorder of articular cartilage, but also an organ failure, involving the whole joint with additional abnormalities especially in bone, ligaments, synovium and joint capsule [6–8]. In particular, the understanding of the role of the nervous system, immune cells and cytokines in the pathophysiology of OA of the hip joint, and their association with the different clinical features of the disease is still limited [4, 9].

Although many studies are available on particular aspects of the role of immune system in pathologic mechanisms in hip OA [10], there are still no consistent reports, and no data is available on the general profile of neuroimmune expression in these patients. Few studies have addressed the cytokine profile in hip OA, and even those, focus only on a small set of cytokines and in a limited range of samples (blood, bone, cartilage or synovial tissue). Moreover, the global picture of hip OA neuroimmune expression is yet to be defined. Therefore, there is a critical need for enlightening on the role of neuroimmune mediators produced at the hip joint in OA patients. This knowledge would be of utmost importance in the ongoing study of pathologic pathways underlying hip OA and an important step in the development of disease-specific modifying therapies.

This systematic review aims to characterize the local and systemic expression of neurochemical and immune biomarkers in patients with hip OA when compared to healthy controls.

## Methods

### Literature search

A systematic search was performed in Pubmed using as main search terms: “neuroimmunity”, “osteoarthritis” and “hip”, and other equivalent terms. The limits used were a) English, French or Portuguese language, b) publication date from 2000 to March 2015, c) studies performed in humans, d) exclusion of reviews, editorials and comments.

### Article selection

**Fig. 1:**
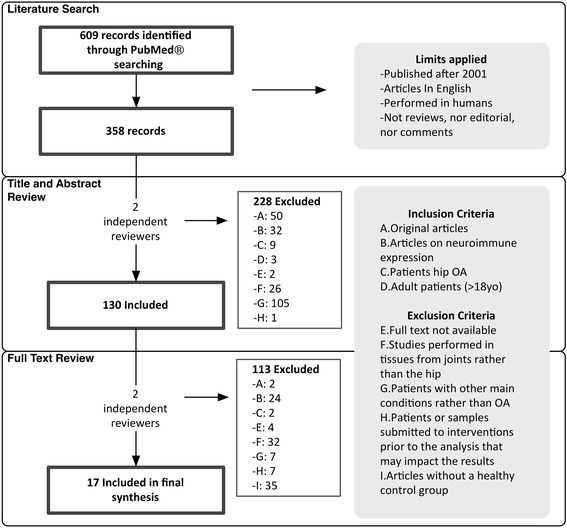
Selection Process

Study selection was conducted in two phases (Fig. 1). In Phase 1, two investigators screened the titles and abstracts independently. If one of them included the abstract, it was allowed into the Phase 2. In Phase 2, full-text articles were analyzed independently, and disagreements were discussed between reviewers. Inclusion criteria were: 1) original data; 2) data on neuroimmune expression; 3) patients with hip OA; 4) adults (>18 years old). Exclusion criteria were: 1) studies performed in tissues other than the hip; 2) participants with known main diseases other than hip OA, e.g. rheumatoid arthritis; 3) patients or samples submitted to intervention prior to the analysis that may influence the results; or 4) absence of a control group. When manuscripts or data were not available, the authors were contacted. One study was excluded because results on cytokine expression were outside the range described by the manufacturer of the technique use and no answer on clarification from the author was received until the end of data analysis.

### Data extraction

Data was extracted using an electronic form developed by the authors and general article information is on Table 1.

**Table 1:** Description of the sample and methods of the studies included and hip OA assessment

Author, year	Sample Size	No		Sample collection	Age		Sex (No of men)		Definition of:	
		*OA*	*C*		*OA*	*C*	*OA*	*C*	*OA*	*C*
Pombo-Suarez, 2009 [20]	22	11	11	Surgery	NI	NI	NI	NI	Clinical, imagiological and histological	Non-OA hip fracture
Koorts, 2012 [16]	26	7	19	Puncture	NI	NI	NI	NI	Clinical and imagiological	Normal X-ray; No inflammation
Granchi, 2006 [12]	128	39	20	Puncture	56 (13)	60 (12)	13	14	Clinical and imagiological	Clinically healthy donnors
Granchi, 2003 [13]	112	22	39	Puncture	60 (3)	52 (3)	8	15	Clinical and imagiological	Clinically healthy donnors
Hashimoto, 2013 [14]	35	7	3	Surgery	52.7 (37–73)^a^	28 (15–44)^a^	5	4	Clinical, imagiological and histological	No tissue degeneration
Abrams, 2014 [10]	34	17	17	Puncture	59.2 (11.9)	38.3 (11.1)	9	5	Clinical and imagiological	Normal X-ray
Pape, 2000 [19]	105	22	20	Puncture	47 (19)	22-26^b^	12	20	Clinical	NI
Dallos, 2009 [11]	NI	NI	NI	NI	NI	NI	NI	NI	Clinical and imagiological	NI
Hulejova, 2007 [7]	85	55	30	Surgery/Puncture	56.4 (10.6)	67.5 (9.5)	9	8	Clinical and imagiological	Healthy; No OA on X-ray; No inflammation
Shi, 2002 [22]	NI	12	10	Surgery	71 (8)	NI	2	4	Clinical and imagiological	No metabolic disease; No OA diagnosis; No OA on surgery
Kumarasinghe, 2012 [17]	NI	5	5	Surgery	67 (12)	81 (6)	0	0	Clinical and imagiological	Non-OA femoral neck fracture?
Lavigne, 2004 [18]	NI	19	11	Surgery	NI	NI	NI	NI	Clinical and imagiological	NI
Saxler, 2007 [5]	6	3	3	Surgery	74	65	0	0	Clinical and imagiological	No pain or degeneration
Sanchez-Sabaté, 2009́ [21]	35	16	11	Surgery	66.9 (7)	42.3 (11.2)	7	7	Clinical and imagiological	No metabolic disease
Takeshita, 2012 [23]	62	50	12	Surgery	61.3 (48–80)^a^	79 (63–90)^a^	4	3	Clinical and imagiological	No clinical or imagiological OA
Verdier, 2005 [24]	9	6	3	Surgery	69-85^b^	76-86^b^	9	9	Clinical and imagiological	Healthy patients with hip fracture
Hopwood, 2007 [15]	35	24	21	Surgery	49-85^b^	43-85^b^	10	10	Clinical and imagiological	No bone disease

All as Mean (Standard Deviation), unless otherwise indicated. *OA* Osteoarthritis, *C* Controls, *NI* No Information. ^a^Mean (range). ^b^Min-Max

The study group was defined as patients with hip OA, upon a diagnosis based on clinical, radiological and/or histological analysis. Controls were defined as healthy patients without OA diagnosis (hip or another).

Studies were grouped based on the technique used for neuroimmune expression measurements, namely: Bead-based multiplex immunoassay, Enzyme-Linked Immunosorbent Assay (ELISA), quantitative real-time polymerase chain reaction (qRT-PCR) or immunostaining; and based on the sample used: synovial fluid, synovial membrane, cartilage, whole blood (blood), serum, immune cells, or bone. In each individual subgroup values were compared between patients with OA and controls (Additional file 1: Table S1).

### Data analysis

Data was gathered on the significance of the comparisons, with a significant statistical value being defined as *p* < 0.05.

Ratios on neuroimmune expression between hip OA and control patients were computed, and a visual illustration with arrows was assembled, grouped by tissue sample (Table 2). If different measurement techniques resulted in different ratios, these were all displayed. A general immune expression pattern was also displayed for each tissue (Table 3).

**Table 2:** Ratios on neuroimmune expression between hip osteoarthritis and controls according to the sample

	SF	SM			Cartilage				Serum	Bone							Immune Cells		Blood	
	Abrams, 2014 [10]	Hulejova, 2007 [7]	Takeshita, 2012 [23]	Saxler, 2005 [5]	Hulejova, 2007 [7]	Hashimoto, 2013 [14]	Verdier, 2003 [24]	Pombo-Suarez, 2009 [20]	Hulejova, 2007 [7]	Hulejova, 2007 [7]	Koorts, 2012 [16]	Shi, 2002 [22]	Kumarasinghe, 2012 [17]	Sanchez-Sabaté, 2009 [21]	Hopwood, 2007 [15]	Lavigne, 2004 [18]	Dallos, 2009 [11]	Granchi, 2003 [13]	Granchi 2006 [12]	Pape, 2000 [19]
BAFF																	↑↑*			
BMP-1														=^b^						
BMP-5															↓↓↓↓					
BMP-6														=^b^						
CGRP			↑↑↑*	↑↑↑↑*																
GM-CSF											↓↓							↑↑↑↑		
ICAM																↑↑				
ICAM-3															↑↑↑↑					
IFN-γ	↑↑										↓↓↓							↑↑↑↑		
IL-10		↑↑↑↑*			↑↑↑↑*				↓↓*	↑↑↑↑*	↓↓↓↓*							↓↓↓↓*		
Il-12											↓↓↓↓									
IL-1Rα	↓↓↓↓																			
IL-1α		↑↑↑↑			↑↑↑*				↓↓↓	↑↑↑↑*										
Il-1β	↓					=^b^					↓↓	=^b^								
IL-2											↓							↑↑↑↑*		
IL-4											↓							↓↓↓↓*		
IL-5											↓↓↓↓									
IL-6	↑↑↑↑										↑	↑*^b^			↑↑↑↑			↓		↑↑↑↑*
IL-8		↑↑↑↑*			↓	=^b^			↓	↑↑↑↑*	↑									
MCP-1	↑																			
MIP-1β	↑↑↑↑																			
NF-Kb			↑↑↑*																	
OPG																			↑↑↑*	
PDGF-ββ	↓↓↓↓																			
PGE-2												↑*^b^								
RANKL																			↓↓	
RANTES	↓↓↓																			
SP				↑↑↑↑*																
TGF-β											↑↑↑*		=^b^							
TGF-β1							↑↑↑	↑↑↑*/↑↑*^a^							↑↑↑↑					
TGF-β2							=	↑↑↑*^a^						↑*^b^						
TGF-β3							↑↑	↑↑↑↑*/↑↑↑*^a^						↑*^b^						
TGF-βR1							↑/=							=^b^	↓↓↓↓					
TNF-α	↓↓↓↓	↑↑↑↑	↑↑↑*		↑↑↑				↑↑*	↑↑↑↑*	↓							↓↓↓↓		↑↑
TuJ-1			↑↑*																	
VEGF	↑↑↑																			
VEGF-b															↑↑↑↑					
VEGF-c															↑↑↑↑					

Ratios were computed as times raised: ↑: 1–1.25; ↑↑: 1.25–1.50;↑↑↑: 1.50–2; ↑↑↑↑: >2; and times decreased: ↓: 1–0.8; ↓↓: 0.8–0.67; ↓↓↓: 0.67–0.5; ↓↓↓↓: <0.5. When values are not available a single arrow (when statistically significant) or = (when non significant) was displayed. Data on statistically significance weren’t available or statistically significant, unless indicated as *p < 0.05

*SF* synovial fluid, *SM* synovial membrane, *Blood* Whole Blood, *hOA* hip Osteoarthritis, *C* Controls. ^a^PCR and Immunohistochemestry. ^b^Values not available

**Table 3:** General pattern of neuroimmune expression

	Sinovial Fluid	Synovial Membrane	Cartilage	Serum	Immune Cells	Blood	Bone
Increased	IFN-γ IL-6 MCP-1 MIP-1β VEGF	IL-10 IL-1α IL-8 TNF- α TGF-β1 TGF-β2 TGF-β3 CGRP NF-Kb TuJ-1 SP	IL-10 IL-1α TNF- α TGF-β1 TGF-β2 TGF-β3	TNF- α	BAFF GM-CSF IFN-γ IL-2	IL-6 OPG TNF- α	BMP-1 BMP-6 ICAM ICAM-3 IL-6 IL-8 PGE-2 TGF-β1 TGF-β2 TGF-β3
Doubtful or Equal			IL-8 Il-1β				Il-1β TGF-β TGF-βR1 TNF- α
Decreased	IL-1Rα Il-1β PDGF-ββ RANTES TNF-α			IL-8	IL-10 IL-4 IL-6 TNF- α	IL-10 IL-1α RANKL	BMP-5 GM-CSF IFN-γ IL-10 IL-12 IL-1α IL-2 IL-4 IL-5 VEGF-b VEGF-c

Analysis presented was based on articles general results. When 2 articles had conflicting data on expression or when comparisons were stated as non-significant data was assigned as doubtful or equal

Only 2 studies were available for the same immune marker when grouped by tissue and technique, and no meta-analysis was performed, since high heterogeneity was predictable.

This systematic review follows the PRISMA recommendations and PRISMA checklist was completed and is available on Additional file 2: Table S2 [11].

## Results

Articles’ search retrieved a total of 609 references. After limits applied 358 were included in the final review. In the first selection phase, 228 articles were excluded, mainly studies with patients with other known conditions than hip OA (Fig. 1). In the second selection phase, all but 4 full-text articles were retrieved and analyzed. Seventeen studies were included in the systematic review [5, 9, 12–26]. Twelve were cross-sectional studies, three cohorts, and one a case-control. Apart from one, all studies primary goal was neuronal and/or immune expression analysis. All were hospital-based studies, with outpatient clinic recruitment. Sample sizes ranged from 6 to 128 participants (Table 1).

Most studies (*n* = 14) based OA diagnosis on clinical and radiological evaluation; one only had a clinical diagnosis, and two also had a histological analysis. Controls definition was highly variable and mostly based on clinical examination and X-ray. Individuals with non-OA hip fracture were used as healthy controls in three studies (Table 1).

Thirty-nine relevant neuroimmune markers were identified from the studies retrieved, and data on the comparison between patients with hip OA and controls was gathered (Additional file 1: Table S1). Their expression was evaluated by five different laboratorial techniques. All but three articles reported in vivo results. The tissue samples studied in the included reports were bone, cartilage, synovial membrane, synovial fluid, whole blood (blood), serum and immune cells (Additional file 1: Table S1).

Six articles did not present numerical values on the analysis performed. Data on general results and significance of the comparisons was gathered when possible and presented (Additional file 1: Table S1).

Fifteen articles reported on immune markers expression and two articles presented results on neurochemical expression. Only one study was available on neuroimmune expression in synovial fluid and in serum. Two were available on immune cells production and blood expression, 3 in synovial membrane, 4 in cartilage and 7 in bone. One article studied more than one tissue, with different techniques (Additional files 1 and 2: Tables S1 and S2).

The following markers showed a different variation on neuroimmune expression in different tissues (Table 2):

- IFN-γ increased in synovial fluid, increased production in immune cells, decreased in bone;
- IL-6, increased in synovial fluid, blood and bone, decreased production in immune cells;
- TNF-α, increased in synovial membrane, cartilage, serum and blood, decreased in synovial fluid and decreased production in immune cells;
- IL-10, increased in synovial membrane and cartilage, decreased in serum and decreased production in immune cells;
- IL-8 is increased in synovial membrane and bone, decreased in serum;
- GM-CSF and IL-2 increased production in immune cells, decreased in bone;
- IL-1α, increased in synovial membrane, cartilage and bone, decreased in serum.

Similar variations in different tissues were recorded for:

- VEGFs, increased in synovial fluid and bone;
-GF-ß isoforms, increased in cartilage and bone.

The collected data was insufficient for quantitative synthesis. Only two studies could be used for just three markers, and so no meta-analysis was performed.

## Discussion

Recent studies showed the joint-specific character signature of the immunity and nervous system activity underlying OA [27]. This is the first systematic review on the neuroimmune expression of patients with hip OA. Few articles were available, and even fewer when sorted among samples studied. Most of the literature regarding hip OA is focused on the immune response and pathological changes of immune mediators. On neurochemical expression, only two articles that meet our inclusion criteria were retrieved. Both showed a tendency to neuropeptide overexpression in synovial membrane [5, 25].

Although this review did not found any specific systematic pattern in each individual tissue, some tendencies on the general neuroimmune expression were observed. Pro-inflammatory cytokines such as IL-6, TNF-α and IL-8 were found local and/or systemically increased in the context of hip OA. Particularly, IL-6 is locally increased in synovial fluid and bone, and also systemically in blood. IL-6 is a pro-inflammatory cytokine, that acts as a stimulator of osteoclast recruitment and bone reabsorption, being related with altered bone metabolism previously described in OA [28, 29]. This goes along with previous works postulating OA as a pro-inflammatory condition [1, 2], and is reinforced by the significant raise of other pro-inflammatory cytokines, such as TNF-α and IL-8. TNF-α was found augmented, both systemic and locally, in synovial membrane, cartilage, bone and blood. It acts both as a mediator of matrix degradation [30] and as an intermediate between immune and nervous system. It is associated with nociceptive response and induces neuronal ingrowth [25, 31]. IL-8 was found increased in both bone and synovial membrane, presenting a pattern of expression similar to IL-1.

IL-10 and IL-4 are known anti-inflammatory cytokines. Previous works reported that they are spontaneously produced in synovial membrane and cartilage [30], probably in an attempt to locally control the inflammatory process [9, 30]. This review supports the findings on IL-10, which is increased in both tissues, but no information was retrieved on IL-4 expression in hip OA patients. Also, the systemic decrease of these anti-inflammatory markers in serum and immune cells, reinforce the ongoing idea of a shift towards a pro-inflammatory state, already reported in hip OA [1, 2].

A local response on cartilage and bone was also observed when analyzing TGF-ß family cytokines. TGF-ß is an inductor of chondrocyte anabolic response and is antagonized by IL-1, that acts as a stimulator of cartilage degradation [18, 32]. Accordingly, both TGF-ß1, − ß2 and -ß3 and IL-1 isoforms were found increased in these tissues. However, no data on the systemic expression of TGF-ß was retrieved, and although one article reported a systemic decrease of IL-1 expression [9], these results were not significant, supporting the theory of a tendency to a local action of IL-1, with no measurable systemic repercussion [33, 34].

RANKL is an osteoclastogenic factor that triggers a cascade of intracellular events, essential to osteoclast activation and differentiation. OPG is a RANKL decoy receptor and limits its biologic activity. Therefore, OPG activation suppresses osteoclast differentiation, inhibits their activation and induces apoptosis [14]. Granchi et al. described an increased expression of OPG in hip OA patients, stating that elevated OPG levels may reflect a protective mechanism of the skeleton to compensate for the osteolytic activity that occurs in severe osteoarthritis [14]. However, in this article, RANKL expression comparisons were not statistically significant [14].

The two articles retrieved on neurochemical expression in patients with hip OA, reported a raise of CGRP, Tuj-1 (neuron-specific class III ß-tubulin) and SP in synovial membrane [5, 25].

The role of nerve fibers and their neurotransmitters in cartilage, subchondral bone, and other joint tissue function and homeostasis is becoming more evident, with reports on the peripheral nervous system involvement in the pathogenesis of disorders such as OA. Suri et al. reported the presence of both sensory (SP- and CGRP-positive) and sympathetic nerve fibers (neuropeptide Y (NPY)-positive) in the articular cartilage, within vascular channels, in both mild and severe stages of knee OA. The exclusively perivascular localization of nerves in the surface layer of articular cartilage implies vascularization as a driving force behind its innervation [35]. Nerve growth is associated with peripheral sensitization. Accordingly, the presence of nerves in structures such as cartilage that are not normally innervated could expose them to chemical stimulation and mechanical stress, explaining why perivascular nerve growth might contribute to the pain mechanisms in OA [8], and particularly in hip OA [5].

Tuj-1 is a neuron-specific class III ß-tubulin that was found in the synovial membrane of patients with hip OA, being absent in the normal controls [31]. The expression of this neurochemical marker occurs after blood vessels and nerve fibres ingrowth from the inflammation of synovial tissue. These inflammatory mechanisms are probably associated with the pain complaints of patients in hip OA [36].

Clinical data from OA patients supports an association between CGRP-immunoreactive fibers and pain [37]. This review retrieved two articles showing an increased expression of this neuronal marker in patients with hip OA, what may also be associated with the pain mechanisms in this condition.

Additionally, in other diseases, such as hip dysplasia, increased levels of SP and CGRP were detected in synovial tissue and fluid and were associated with catabolic and pro-inflammatory effects [38]. SP, also found increased in hip OA patients, was implicated in the modulation of the physiological metabolism of chondrocytes and cartilage homeostasis, with catabolic effects on articular cartilage during OA [39].

This is corroborated by other works stating the importance of these peptides in modulation of the inflammatory process and in signaling of pain in OA [40], being increased in all stages of inflammation [41].

Overall, our review goes along with previous reports on OA, with no relevant differences found between hip OA neuroimmune expression and the one reported in general OA patients (24). A recent review on immune expression showed a tendency towards an overexpression of cytokines in patients with OA, with a role for inflammation in the disease severity and progression [4]. Our review confirms these results, showing an overall increase of cytokine expression in OA and reinforcing the idea of a link between a deregulated function of the neuroimmune system and the development and perpetuation of the disease [4]. Nevertheless, no specific systematic pattern on neurochemical changes in OA was found. This work brings light on the need to further studies on the neuroimmune axis in joint-related conditions, as its role is yet to be clearly defined.

The individual methods found in the retrieved works were heterogeneous. Some studies provided no definition for the control group, and others used patients with proximal femur fractures as controls. Even if stated by individual authors that no OA was observed when this last group was used as control, one cannot exclude both the influence of the fracture itself and the possible influence of concomitant milder undiagnosed forms of OA. Patients affected with proximal femur fractures are elderly subjects, which can be affected by milder forms of hip OA, with local and systemic biochemical changes before the time a radiological diagnosis of hip OA is made [42]. Also, fractures, due to the inherent aggression, are associated with both a local and a systemic biochemical response, with an increased inflammatory response [43]. Both undiagnosed hip OA and the fracture-associated inflammatory reaction can lead to an underestimation of the neuroimmune activation in patients with hip OA, which represents an import bias in these comparisons [42]. Furthermore, it is known that expression of individual molecules changes along OA progression. Since many articles do not state the stage of the disease in which each sample collected, one cannot reliably assure that the comparisons established refer to similar timing of the disease, what can have a major impact in the results presented. Lastly, the revised studies used different methodologies to assess the targets across the analyzed tissues. Thus, the sensitivity (e.g. ELISA vs Lumina(R) Multiplex) and the evaluated form of the target (e.g. mRNA vs. protein) limits the reliability of a molecular hip OA profile [44].

Our study has some limitations. Firstly, a quantitative data synthesis by meta-analysis was not possible, as only a few studies were available in each molecule expression for a specific tissue, with different outcome measurements. Also, most studies included a small number of patients, a problem also stated in a previous review, that implies a need for future confirmation of these data in additional studies or in larger cohorts [4]. Some studies reported results only from qualitative outcomes, and others do not report the significance of their quantitative results. This further impacts our ability to properly analyze their results. As stated before, one study was even excluded since no reliable data was provided.

Nevertheless, this is the first available systematic review on neuroimmune expression in human patients with OA, and especially with hip OA, without any limitation for sample size, age group, sex or type of sample studied. Two blinded reviewers analyzed the articles in each review phase, diminishing the risk of selection bias. Only 4 full-texts were not available, with high full-text article retrieval rate.

Future studies with strictly defined rules on control and patient selection, as well as disease progression stage, demographic characteristics of samples, sample collection, processing and analysis are needed. As stated by previous reports, a correlation with clinical features of the disease may also be a valuable resource in future strategies for directing therapy investigations [4]. Few information was available on neurochemical activity in these patients, and in what comes to immune system, most studies focus only on classic cytokines. New and more information on different and recent found targets are required [2, 11, 45]. Also, and particularly for hip OA, there is a need to study the role of neuroimmune expression on the functional impairment and pain levels reported by these patients. It is also important to have a previously defined set of molecules with central roles in this disease, to have a more uniform report among future works. Larger samples are needed to provide more reliable results.

## Conclusions

This is the first systematic review available on neuroimmune expression on hip OA and highlights a key role of inflammation in both disease maintenance and progression. It is associated with an overall upregulation of the neuroimmune system, confirming previous reports on a deregulated balance between pro and anti-inflammatory cytokines, both locally and systemically, impacting cartilage and bone remodelling. This review enhances the importance of further studies with a simultaneous assessment on immune and neurochemical expression in these patients, following clearly defined criteria and similar methodological strategies.

## Additional files

Additional file 1: Table S1. Comparison of neuroimmune expression between hip OA and controls in the included studies. Values are grouped by assessment technique and tissue studied. Articles are presented as references for space purposes. All articles were in vivo, except for the ones marked. Data is presented with 2 decimal cases, as Mean (Standard Deviation), otherwise indicated. *Median (25th–75th). ^#^ng/g. ^&^pg/mL. ^¥^fibers/cm^2^. ^%^In vitro. ^$^Bead based multiplex immunoassay. Art.:Article. SF: synovial fluid. SM: synovial membrane. C: Cartilage. Bo: Bone. Bl: Whole Blood. S: Serum. IC: Immune Cells. hOA: hip Osteoarthritis. C: Controls. NI: No information. (DOCX 48 kb)

Additional file 2: Table S2. PRISMA Checklist. (DOC 62 kb)

## Abbreviations

Blood: Whole blood
ELISA: Enzyme-Linked Immunosorbent Assay
OA: Osteoarthritis
qRT-PCR: quantitative real-time polymerase chain reaction
