# Supplementary material for: Neuroimmune expression in hip osteoarthritis: a systematic review
Source: BMC Musculoskelet Disord. 2017 Sep 11;18:394. doi: 10.1186/s12891-017-1755-2 (PMC5594449; doi:10.1186/s12891-017-1755-2)
Supplement: Supplementary file 1 — Comparison of neuroimmune expression between hip OA and controls in the included studies. Values are grouped by assessment technique and tissue studied. Articles are presented as references for space purposes. All articles were in vivo, except for the ones marked. Data is presented with 2 decimal cases, as Mean (Standard Deviation), otherwise indicated. *Median (25th–75th). #ng/g. &pg/mL. ¥fibers/cm2. %In vitro. $Bead based multiplex immunoassay. Art.:Article. SF: synovial fluid. SM: synovial membrane. C: Cartilage. Bo: Bone. Bl: Whole Blood. S: Serum. IC: Immune Cells. hOA: hip Osteoarthritis. C: Controls. NI: No information. (DOCX 48 kb) [file 12891_2017_1755_MOESM1_ESM.docx]

| **Technique** | **Sample** | **Art.** | **Group** | **BAAF** | **BMP-1** | **BMP-5** | **BMP-6** | **CGRP** | **GM-CSF** | **ICAM** | **ICAM-3** | **IFN-γ** | **IL-10** | **Il-12** | **IL-1Rα** | **IL-1α** | **Il-1β** | **IL-2** | **IL-4** | **IL-5** | **IL-6** | **IL-8** | **MCP-1** | **MIP-1β** | **NF-Kb** | **OPG** | **PDGF-ββ** | **PGE-2** | **RANKL** | **RANTES** | **SP** | **TGF-β** | **TGF-β1** | **TGF-β2** | **TGF-β3** | **TGF-βR1** | **TNF-α** | **TuJ-1** | **VEGF** | **VEGF-b** | **VEGF-c** |
| --- | --- | --- | --- | --- | --- | --- | --- | --- | --- | --- | --- | --- | --- | --- | --- | --- | --- | --- | --- | --- | --- | --- | --- | --- | --- | --- | --- | --- | --- | --- | --- | --- | --- | --- | --- | --- | --- | --- | --- | --- | --- |
| **Bead-based multiplex immunoasssay (μg/mL)** | **SF** | Abrams, 2014 [10] | hOA |  |  |  |  |  |  |  |  | 199.12 (188.9) |  |  | 1108.07 (1032.1) |  | 11.36 (11)^&^ |  |  |  | 182.5 (93.4) |  | 44.04 (25) | 26 (13) |  |  | 56.87 (22) |  |  | 188.08 (49.4) |  |  |  |  |  |  | 79.22 (70.6) |  | 258.17 (60.2) |  |  |
|  |  |  | C |  |  |  |  |  |  |  |  | 151.06 (123.2) |  |  | 4026.03 (3183.3) |  | 13.12 (11.9)^&^ |  |  |  | 61.3 (30.4) |  | 35.93 (12.1) | 9 (4) |  |  | 270.71 (151) |  |  | 341.95 (85.5) |  |  |  |  |  |  | 198.25 (140.7) |  | 140.52 (38.2) |  |  |
|  | **SM** | Hulejova,  2007^#^ [7] | hOA |  |  |  |  |  |  |  |  |  |  |  |  |  |  |  |  |  |  | 2.4 (2.8) |  |  |  |  |  |  |  |  |  |  |  |  |  |  | 1.9 (0.5) |  |  |  |  |
|  |  |  | C |  |  |  |  |  |  |  |  |  |  |  |  |  |  |  |  |  |  | 0.4 (0.3) |  |  |  |  |  |  |  |  |  |  |  |  |  |  | 0.8 (0.1) |  |  |  |  |
|  | **C** | Hulejova,  2007 [7] | hOA |  |  |  |  |  |  |  |  |  |  |  |  |  |  |  |  |  |  | 13.5 (4.1) |  |  |  |  |  |  |  |  |  |  |  |  |  |  | 2.5 (0.5) |  |  |  |  |
|  |  |  | C |  |  |  |  |  |  |  |  |  |  |  |  |  |  |  |  |  |  | 16.4 (9.2) |  |  |  |  |  |  |  |  |  |  |  |  |  |  | 1.5 (0.3) |  |  |  |  |
|  | **S** | Hulejova,  2007 [7] | hOA |  |  |  |  |  |  |  |  |  |  |  |  |  |  |  |  |  |  | 9.2 (1.4) |  |  |  |  |  |  |  |  |  |  |  |  |  |  | 11.8 (4) |  |  |  |  |
|  |  |  | C |  |  |  |  |  |  |  |  |  |  |  |  |  |  |  |  |  |  | 9.4 (1.1) |  |  |  |  |  |  |  |  |  |  |  |  |  |  | 8.8 (0.7) |  |  |  |  |
|  | **Bo** | Hulejova,  2007^#^ [7] | hOA |  |  |  |  |  |  |  |  |  |  |  |  |  |  |  |  |  |  | 11.7 (5) |  |  |  |  |  |  |  |  |  |  |  |  |  |  | 2.3 (0.6) |  |  |  |  |
|  |  |  | C |  |  |  |  |  |  |  |  |  |  |  |  |  |  |  |  |  |  | 1.1 (0.5) |  |  |  |  |  |  |  |  |  |  |  |  |  |  | 0.4 (0.01) |  |  |  |  |
|  |  | Koorts,  2012 [16] | hOA |  |  |  |  |  | 1.96 (7.4)^&^ |  |  | 0.1 (0)^&^ | 2.17 (3.5)^&^ | 0.63 (0.6)^&^ |  |  | 0.37 (0.9) ^&^ | 4.07 (10)^&^ | 1.24 (3.2)^&^ | 1.11 (5.8)^&^ | 4.37 (3.4)^&^ | 16.38 (9.6)^&^ |  |  |  |  |  |  |  |  |  | 16.69 (6.2)^&^ |  |  |  |  | 2.05 (1.5)^&^ |  |  |  |  |
|  |  |  | C |  |  |  |  |  | 2.58 (10.8)^&^ |  |  | 0.18 (0.4)^&^ | 4.56 (3.9)^&^ | 2.73 (5)^&^ |  |  | O.49 (1) ^&^ | 5.04 (11.9)^&^ | 1.48 (2.3)^&^ | 2.23 (4.4)^&^ | 3.56 (5.6)^&^ | 14.2 (19.4)^&^ |  |  |  |  |  |  |  |  |  | 8.6 (6.8)^&^ |  |  |  |  | 2.29 (1.1)^&^ |  |  |  |  |
| **ELISA (pg/mL) – mean (sd)** | **SM** | Hulejova,  2007^#^ [7] | hOA |  |  |  |  |  |  |  |  |  | 1.5 (0.6) |  |  | 0.4 (0.1) |  |  |  |  |  |  |  |  |  |  |  |  |  |  |  |  |  |  |  |  |  |  |  |  |  |
|  |  |  | C |  |  |  |  |  |  |  |  |  | 0.2 (0.02) |  |  | 0.2 (0.04) |  |  |  |  |  |  |  |  |  |  |  |  |  |  |  |  |  |  |  |  |  |  |  |  |  |
|  | **C** | Hulejova,  2007 [7] | hOA |  |  |  |  |  |  |  |  |  | 3.3  (1) |  |  | 0.8 (0.3) |  |  |  |  |  |  |  |  |  |  |  |  |  |  |  |  |  |  |  |  |  |  |  |  |  |
|  |  |  | C |  |  |  |  |  |  |  |  |  | 0.1 (0.01) |  |  | 0.5 (0.1) |  |  |  |  |  |  |  |  |  |  |  |  |  |  |  |  |  |  |  |  |  |  |  |  |  |
|  | **IC** | Dallos,  2009^%^ [11] | hOA | 685.0 (574.8-809.3)* |  |  |  |  |  |  |  |  |  |  |  |  |  |  |  |  |  |  |  |  |  |  |  |  |  |  |  |  |  |  |  |  |  |  |  |  |  |
|  |  |  | C | 527.3 (462.1-568.0)* |  |  |  |  |  |  |  |  |  |  |  |  |  |  |  |  |  |  |  |  |  |  |  |  |  |  |  |  |  |  |  |  |  |  |  |  |  |
|  |  | Granchi,  2003^%^ [13] | hOA |  |  |  |  |  | 2335 (869) |  |  | 1925 (849) | 86 (64) |  |  |  |  | 1692 (400) | 0.1 (0.001) |  | 3008 (894) |  |  |  |  |  |  |  |  |  |  |  |  |  |  |  | 850 (206) |  |  |  |  |
|  |  |  | C |  |  |  |  |  | 963 (145) |  |  | 861 (266) | 434 (141) |  |  |  |  | 498 (103) | 73 (18) |  | 3027 (535) |  |  |  |  |  |  |  |  |  |  |  |  |  |  |  | 1949 (972) |  |  |  |  |
|  | **Bl** | Granchi,  2006 [12] | hOA |  |  |  |  |  |  |  |  |  |  |  |  |  |  |  |  |  |  |  |  |  |  | 4044 (1932) |  |  | 1115 (1074) |  |  |  |  |  |  |  |  |  |  |  |  |
|  |  |  | C |  |  |  |  |  |  |  |  |  |  |  |  |  |  |  |  |  |  |  |  |  |  | 2586 (1082) |  |  | 1606 (1245) |  |  |  |  |  |  |  |  |  |  |  |  |
|  |  | Pape, 2000  [19] | hOA |  |  |  |  |  |  |  |  |  |  |  |  |  |  |  |  |  | 46 (16) |  |  |  |  |  |  |  |  |  |  |  |  |  |  |  | 0.1 (0.05) |  |  |  |  |
|  |  |  | C |  |  |  |  |  |  |  |  |  |  |  |  |  |  |  |  |  | 12 (18) |  |  |  |  |  |  |  |  |  |  |  |  |  |  |  | 0.07 (0.06) |  |  |  |  |
|  | **S** | Hulejova,  2007 [7] | hOA |  |  |  |  |  |  |  |  |  | 17.5 (1.9) |  |  | 4.1 (0.5) |  |  |  |  |  |  |  |  |  |  |  |  |  |  |  |  |  |  |  |  |  |  |  |  |  |
|  |  |  | C |  |  |  |  |  |  |  |  |  | 22.0 (2.1) |  |  | 7.3 (0.6) |  |  |  |  |  |  |  |  |  |  |  |  |  |  |  |  |  |  |  |  |  |  |  |  |  |
|  | **Bo** | Hulejova,  2007^#^ [7] | hOA |  |  |  |  |  |  |  |  |  | 1.2 (0.6) |  |  | 0.2 (0.05) |  |  |  |  |  |  |  |  |  |  |  |  |  |  |  |  |  |  |  |  |  |  |  |  |  |
|  |  |  | C |  |  |  |  |  |  |  |  |  | 0.1 (0.01) |  |  | 0.05 (0.01) |  |  |  |  |  |  |  |  |  |  |  |  |  |  |  |  |  |  |  |  |  |  |  |  |  |
|  |  | Shi, 2002^%^  [12] | hOA |  |  |  |  |  |  |  |  |  |  |  |  |  | NI |  |  |  | NI |  |  |  |  |  |  | NI |  |  |  |  |  |  |  |  |  |  |  |  |  |
|  |  |  | C |  |  |  |  |  |  |  |  |  |  |  |  |  | NI |  |  |  | NI |  |  |  |  |  |  | NI |  |  |  |  |  |  |  |  |  |  |  |  |  |
|  |  | Kumarasinghe, 2012^%^  [17] | hOA |  |  |  |  |  |  |  |  |  |  |  |  |  |  |  |  |  |  |  |  |  |  |  |  |  |  |  |  | NI |  |  |  |  |  |  |  |  |  |
|  |  |  | C |  |  |  |  |  |  |  |  |  |  |  |  |  |  |  |  |  |  |  |  |  |  |  |  |  |  |  |  | NI |  |  |  |  |  |  |  |  |  |
| **qRT-PCR (fold-change)** | **C** | Pombo-Suarez, 2009 [20] | |  |  |  |  |  |  |  |  |  |  |  |  |  |  |  |  |  |  |  |  |  |  |  |  |  |  |  |  |  | 1.6 | 1.7 | 2.3 |  |  |  |  |  |  |
|  |  | Hashimoto, 2013 [14] | |  |  |  |  |  |  |  |  |  |  |  |  |  | NI |  |  |  |  | NI |  |  |  |  |  |  |  |  |  |  |  |  |  |  |  |  |  |  |  |
|  | **Bo** | Sanchez-Sabaté, 2009 [21] | |  | NI |  | NI |  |  |  |  |  |  |  |  |  |  |  |  |  |  |  |  |  |  |  |  |  |  |  |  |  |  | NI | NI | NI |  |  |  |  |  |
| **Microarray (fold-change)** | **Bo** | Hopwood, 2007 [15] | |  |  | -10.31 |  |  |  |  | 3.30 |  |  |  |  |  |  |  |  |  | 2.59 |  |  |  |  |  |  |  |  |  |  |  | 2.74 |  |  | -4.92 |  |  |  | 3.24 | 5.30 |
| **Immunostaining (median range)**  (all  imunohistochemestry except article 12 - immunocitology) | **SM** | Takeshita, 2012 [23] | hOA |  |  |  |  | 54% |  |  |  |  |  |  |  |  |  |  |  |  |  |  |  |  | 68% |  |  |  |  |  |  |  |  |  |  |  | 58% | 46% |  |  |  |
|  |  |  | C |  |  |  |  | 0% |  |  |  |  |  |  |  |  |  |  |  |  |  |  |  |  | 0% |  |  |  |  |  |  |  |  |  |  |  | 0% | 0% |  |  |  |
|  |  | Saxler,  2007 [5] | hOA |  |  |  |  | 15.6  (14.3-18.8)^¥^ |  |  |  |  |  |  |  |  |  |  |  |  |  |  |  |  |  |  |  |  |  |  | 8.2 (6.1-9.1)^¥^ |  |  |  |  |  |  |  |  |  |  |
|  |  |  | C |  |  |  |  | 5.7  (3.9-7.2)^¥^ |  |  |  |  |  |  |  |  |  |  |  |  |  |  |  |  |  |  |  |  |  |  | 3.2 (1.8-4.2)^¥^ |  |  |  |  |  |  |  |  |  |  |
|  | **Bo** | Lavigne,  2004^%^ [18] | hOA |  |  |  |  |  |  | 48.6% |  |  |  |  |  |  |  |  |  |  |  |  |  |  |  |  |  |  |  |  |  |  |  |  |  |  |  |  |  |  |  |
|  |  |  | C |  |  |  |  |  |  | 37.5% |  |  |  |  |  |  |  |  |  |  |  |  |  |  |  |  |  |  |  |  |  |  |  |  |  |  |  |  |  |  |  |
|  | **C** | Pombo-Suarez, 2009 [20] | hOA |  |  |  |  |  |  |  |  |  |  |  |  |  |  |  |  |  |  |  |  |  |  |  |  |  |  |  |  |  | +++ | ++ | ++ |  |  |  |  |  |  |
|  |  |  | C |  |  |  |  |  |  |  |  |  |  |  |  |  |  |  |  |  |  |  |  |  |  |  |  |  |  |  |  |  | ++ | + | + |  |  |  |  |  |  |
|  |  | Verdier,  2007 [24] | hOA |  |  |  |  |  |  |  |  |  |  |  |  |  |  |  |  |  |  |  |  |  |  |  |  |  |  |  |  |  | +++ | ± | +++ | ++ |  |  |  |  |  |
|  |  |  | C |  |  |  |  |  |  |  |  |  |  |  |  |  |  |  |  |  |  |  |  |  |  |  |  |  |  |  |  |  | +/++ | ± | ++ | +/++ |  |  |  |  |  |

**Additional file 1: Table S1. Comparison of neuroimmune expression between hip OA and controls in the included studies**

Values are grouped by assessment technique and tissue studied. Articles are presented as references for space purposes. All articles were in vivo, except for the ones marked. Data is presented with 2 decimal cases, as Mean (Standard Deviation), otherwise indicated.

*Median (25^th^-75^th^). ^#^ng/g. ^&^pg/mL. ^¥^fibers/cm^2^. ^%^In vitro. ^$^Bead based multiplex immunoassay. Art.:Article. SF: synovial fluid. SM: synovial membrane. C: Cartilage. Bo: Bone. Bl: Whole Blood. S: Serum. IC: Immune Cells. hOA: hip Osteoarthritis. C: Controls. NI: No information.
